# Hydroxychloroquine non-availability during COVID-19 pandemic and its relation to anxiety level and disease activity in rheumatoid arthritis and lupus patients: a cross-sectional study

**DOI:** 10.1186/s43166-022-00117-1

**Published:** 2022-03-10

**Authors:** Mohammed Hassan Abu-Zaid, Hany M. Aly, Abdelhafeez Moshrif, Doaa E. Abdeldaim, Nehal El-Ghobashy

**Affiliations:** 1 Rheumatology and Rehabilitation, Faculty of Medicine, Tanta University, Tanta, Egypt; 2 Rheumatology and Rehabilitation, Faculty of Medicine, Al-Azhar University (Cairo), Al-Mokhayam Al-daem St. Nasr City, Cairo, Egypt; 3 Rheumatology Department, Faculty of Medicine, Al-Azhar University (Assiut), 71524, Al-Azhar University Square, King Faysal, Assiut, Egypt; 4 Faculty of Medicine, Tanta University, Tanta, Egypt; 5 Faculty of Medicine, Cairo University, Cairo, Egypt

## Abstract

**Background:**

During COVID-19 disease era, there is an accelerated demand for hydroxychloroquine since it was suggested by some centers as potential therapy for COVID-19 which has led to diminished supplies for patients with rheumatic disease and which carried unexpected risk for disease flare particularly in patients with systemic lupus erythematosus and rheumatoid arthritis. The purpose of the work is to detect the effect of HCQ shortage in patients with RA and SLE on anxiety and disease activity.

**Results:**

A total of 320 patients were divided into two groups: group 1**—**216RA patients with mean age 45.5 ± 9.59 years, disease duration 43.4 ± 25.6 months with female predominance (62.5%). Group 2—104 SLE patients with mean33.4 ± 7.9 age years, disease duration 52.1 ± 34.6 months with female predominance (85.6%). HCQ shortage occurred in 174 RA patients (80.5%) and 76 lupus patients (73.1%). Despite HCQ shortage, there were no significant change in disease activity of RA (using CDAI) and SLE (using SELENA) *p* = 0.063 and 0.064 respectively before and after HCQ shortage. Anxiety level was higher in patients who were exposed to HCQ shortage in both groups (SLE *p* 0.0058 and RA *p* 0.0044) when we compared them to those without HCQ shortage.

**Conclusion:**

In most patients with RA and SLE, the COVID-19 pandemic resulted in a HCQ scarcity, with no effect on disease activity. Anxiety was found to be associated with HCQ shortage in both diseases.

## Background

Rheumatoid arthritis (RA) is a chronic inflammatory autoimmune condition that affects multiple body cells and has a high disease morbidity and mortality rate [1]. It is affected by degradation of autoreactive T cell immune tolerance and/or suppressive T cell dysfunction [2].

Systemic lupus erythematosus (SLE) is a multi-system autoimmune disorder that contributes significantly to global morbidity and mortality. It primarily affects women with a ratio of 10 to 1 between women and men during their reproductive life [3].

Hydroxychloroquine (HCQ) and chloroquine (CQ) are widely used anti-malarial drugs that elicit immunomodulatory effects and have a variety of mechanisms to aggregate in the lysosome and autophagosome of cells, and to impact antigen presentation and major histocompatibility cells (MHCs) class II; they also inhibit the development of pro-inflammatory cytokines [4].

Throughout the globe, fears of HCQ supply constraints for rheumatic patients are increasing as an implication of the overwhelming science and public interest for HCQ as a possible COVID-19 medication, and this affects rheumatic patients who use HCQ in their therapeutic management [5].

Through COVID-19 infection, several studies of insomnia, psychiatric pain, anxiety, depression, and signs of traumatic stress in the common public in Asia and Europe have been reported [6–9]. Salari et al. stated in their systematic review and meta-analysis that the incidence of stress, anxiety, and depression is 29.6%, 31.9%, and 33.7%, respectively [9].

The aim of this study is to detect the influence of HCQ shortage on disease activity and anxiety in patients with RA and SLE during COVID-19 pandemic.

## Methods

### Study design

This cross-sectional multicenter study included 216 patients with rheumatoid arthritis (fulfilling the 2010 American College of Rheumatology (ACR) Classification criteria for Rheumatoid Arthritis) [10] and 104 lupus patients (fulfilling the 2012 SLICC classification criteria for SLE) [11] receiving Hydroxychloroquine were included in this cross-sectional multicenter study. Patients were recruited from outpatient clinics of rheumatology and rehabilitation departments of our university hospitals.

Patients who did not have HCQ in their treatment regimen and patients with known psychological disorders or severe debilitating disease were excluded from the study.

In this study, all participants were subjected to history taking and clinical examination. The laboratory investigations of relevance were assessed including erythrocyte sedimentation rate (ESR) and autoimmune profile of importance.

Clinical disease assessment data were recorded twice:

1. Before HCQ shortage (from data of patients recorded in their medical files)
2. Three months after HCQ shortage.

Methods of assessment of the patients:

1. Assessment of incidence of HCQ shortage by asking the patient the following questions:

Have you faced any difficulty in finding HCQ? If yes.

Have you stopped HCQ because of its shortage?

1. Clinical assessment of disease activity was done using the following:

- Clinical Disease Activity Index (CDAI) [12].
- SLEDAI-SLE disease activity index (SELENA modification) [13].

2- Assessment of anxiety by Hamilton anxiety rating scale (14 questions with a total score range of 0–56) [14].

### Statistical analysis

Statistical Package for Social Sciences (SPSS) (version 23.0, IBM, Armonk, NY) was used to analyze the data of the study. Descriptive presentations were done for all variables of the study and comparison between groups was done by using chi-square test (*χ*^2^). Quantitative variables were presented by mean and standard deviation and comparison between them was done by independent *t* test. Comparison between data before and after COVID-19 in the same group was done by paired *t* test. Level of significance was at *p* ≤ 0.05.

## Results

This study included 216 RA patients and 104 lupus patients. Most of patients were female and with mean age of 45.5 ± 9.59 years and 33.4 ± 7.9 years respectively. Most of patients were married with disease duration 43.4 ± 25.6 months and 52.1 ± 34.6 months respectively. Most of RA patients received methotrexate and low dose corticosteroids, while most of SLE patients received corticosteroids and either azathioprine or mycophenolate mofetyl. HCQ shortage occurred in 174 RA patients (80.5%), and 76 lupus patients (73.1%), with mean duration of shortage 103.2 ± 49.4 and 88.6 ± 23.7 days respectively. The demographic and clinical data of both groups were represented in Table 1.

**Table 1:** Demographic and clinical data of both groups

	Group I: RA patients (216)	Group II: SLE patients (104)
**Age (years)** mean ± SD	45.5 ± 9.59	33.4 ± 7.9
**Sex (M/F)**	81/135	15/89
**Residence**		
Urban/rural	108/108	60/44
**Marital status**		
Married/not married	207/9	94/10
**Disease duration** (months) Mean ± SD	43.4 ± 25.6	52.1 ± 34.6
**Disease presentation (number of patients)**		
Articular	216	60
Cutaneous	0	98
Serositis	4	56
Nephritis	0	31
Neuropsychiatric	0	4
Cardiac	0	7
Hematological	0	32
Vasculitis	1	3
APS	0	6
**Comorbidities (number of patients)**		
Hypertension	13	10
Diabetes	8	3
Hyperlipidemia	6	4
Chronic kidney disease	0	2
Interstitial lung disease	2	1
Thyroid dysfunction	6	8
**HCQ shortage** (Y/N)	174/42	76/28
Duration of shortage (days) mean ± SD	103.2 ± 49.4	88.6 ± 23.7
**Lab findings** ***N*** **(%)**		
RF	147 (68%)	5 (4.8%)
Anti-CCP	174 (80.5%)	0
ANA	12 (5.5%)	104 (100%)
Anti-dsDNA	0	84 (80.8%)
Hypocomplementemia	0	46 (44.2%)
**Treatment received (number of patients)**		
Methotrexate	135	3
Leflunomide	96	0
Azathioprine	4	56
Mycophenolate mofetyl	0	45
Cyclophosphamide	0	4
Corticosteroids	125	88
Biological therapy	0	0

*HCQ* hydroxychloroquine, *RF* rheumatoid factor, anti-CCP, anti-cyclic citrullinated peptide, *ANA* anti-nuclear antibody

By using dependent *t* test to compare disease activity before and after HCQ shortage, there were no significant changes in disease activity of RA (using CDAI), and SLE (using SELENA) *p* = 0.063 and 0.064 respectively. This was explained in Table 2.

**Table 2:** Disease activity before and after HCQ shortage

	Before shortage	After shortage	Paired ***t*** test	***P*** value
**CDAI in RA patients**	11.47 ± 5.62	12.51 ± 5.97	1.86	0.063
**SELENA in SLE patients**	4.86 ± 3.78	5.88 ± 4.11	1.86	0.06**4**

*CDAI* clinical disease activity index, *RA* rheumatoid arthritis, *SELENA* safety of estrogens in systemic lupus erythematosus national assessment, *SLE* systemic lupus erythematosus

*Significant *P* value if *p* < 0.05

Anxiety level was higher in patients who exposed to HCQ shortage in both groups when we compared them to those unexposed to HCQ shortage, and this was explained in details in Tables 3 and 4.

**Table 3:** Anxiety “Hamilton Anxiety Rating Scale” in RA patients during COVID-19 outbreak

	With HCQ shortage (174)	Without HCQ shortage (42)	***P*** value
**Anxiety level**	24.31 ± 9.6	19.73 ± 7.45	0.0044*
**Mild**	33 (18.9%)	16 (38.1%)	0.031*
**Mild to moderate**	58 (33.3%)	13 (31%)	
**Moderate to severe**	56 (32.3%)	11 (26.2%)	
**Severe**	27 (15.5%)	2 (4.7%)	

*HCQ* hydroxychloroquine

*Significant *P* value if *p* < 0.05

**Table 4:** Anxiety “Hamilton Anxiety Rating Scale” in SLE patients during COVID-19 outbreak

	With HCQ shortage (76)	Without HCQ shortage (28)	***P*** value
**Anxiety level**	28.61 ± 10.83	22.11 ± 9.57	0.0058*
**Mild**	5 (6.6%)	8 (28.6%)	0.006*
**Mild to moderate**	22 (28.9%)	11 (39.3%)	
**Moderate to severe**	33 (43.4%)	6 (21.4%)	
**Severe**	16 (21.1%)	3 (10.7%)	

*SLE* systemic lupus erythematosus, *HCQ* hydroxychloroquine

*Significant *P* value if *p* < 0.05

There was significant positive correlation between anxiety level and CDAI in group I (*P* = 0.006); also, there was significant positive correlation between anxiety level and SELSNA in group II (*P* = 0.009) **(**Table 5).

**Table 5:** Correlation between anxiety "Hamilton Anxiety Rating Scale" and disease activity measurements

	***r***	***p***
**CDAI**	0.186	0**.**006*
**SELENA**	0.254	0.009*

*CDAI* clinical disease activity index, *SELENA* safety of estrogens in systemic lupus erythematosus national assessment

*Significant *P* value if *p* < 0.05

From the logistic regression model to evaluate the factors associated with anxiety**,** we observed that anxiety was significantly associated with HCQ shortage, the duration of shortage, CDAI, and SELENA. Table 6 mentions these associations in details.

**Table 6:** Relation between anxiety “Hamilton Anxiety Rating Scale” and different variables

	OR	***P***
**Group I (RA)**		
**Age**	0.98	0.13
**Sex**	1.1	0.17
**Marital state**	1.5	0.18
**Disease duration**	0.92	0.12
**HCQ stoppage**	1.9	0.03*
**Duration of stoppage**	3.8	0.017*
**CDAI**	1.09	0.01
**Group II (SLE)**		
**Age**	2.1	0.02*
**Sex**	0.45	0.09
**Marital state**	0.9	0.17
**Disease duration**	0.98	0.06
**HCQ stoppage**	1.6	0.03*
**Duration of stoppage**	4.2	0.04*
**SELENA**	1.1	0.02*

*CDAI* clinical disease activity index, *RA* rheumatoid arthritis, *SELENA* safety of estrogens in systemic lupus erythematosus national assessment, *SLE* systemic lupus erythematosus, *HCQ* hydroxychloroquine

Logistic regression test

*Significant *P* value if *p* < 0.05

## Discussion

Over the decades, HCQ remains the pillar pharmacotherapy in treatment of variety of rheumatic disease. Patients with SLE who usually take HCQ experience improvement in overall disease activity, beyond lupus, patients with rheumatic disease who take HCQ advantage from improvement in their thrombotic risk, lipid profile, and glycemic index [15]. For a long time, the 1995 HERA study [16] demonstrated that it has a significant benefit on synovitis, pain, and physical disability in patients with early-stage RA.

Across the globe, concerns of hydroxychloroquine (HCQ) supply shortages for patients with rheumatic disease are growing [17] in part because of the immense scientific and public eagerness for HCQ as a potential COVID-19 therapy [18].

The demand surge for HCQ extremely influenced access to this drug among patients with SLE and RA [19]. The purpose of this study is to explore the effect of HCQ shortage on Egyptian SLE and RA patients.

In our study, we enrolled 320 patients from different four rheumatology departments representing different regions in Egypt (Cairo, Delta, and Upper Egypt). Our patients in RA group and SLE group experienced hydroxychloroquine shortage 80.5%and 73% respectively due to drug unavailability with no significant changes in disease activity of RA (using CDAI), and SLE (using SELENA) *p* = 0.063 and 0.064 respectively. In a study conducted by Abualfadl E et al., 41.8% of rheumatoid arthritis patients reported difficulty to obtain HCQ and about 40.7% experienced flare of their disease [20]. This difference could be attributed to difference in sample size between both studies. As regards SLE, our results agree with a report published by EULAR in 2021 which showed that 55% of patients with SLE experienced HCQ shortage during COVID-19 pandemic [21]. In a study by Gheita et al. on Egyptian patients in 2020, 90% of SLE patients and 50% of RA patients were exposed to HCQ non-availability [22].

Lupus patients already have exceptionally high levels of comorbid anxiety and depression, which are related to worse lupus outcomes and higher side-effect burden [23]. Further, there is mounting proof that psychological stress, as seen in depression and anxiety, may contribute to inflammation in SLE. In this study, anxiety level was higher in SLE patients who were exposed to HCQ shortage (*p*: 0.009). Although we found positive correlation between anxiety level and SELENA (*p*: 0.0058). In agreement to our results, Pawluk et al. followed 41 female SLE patients for 6 months and found that higher levels of daily stress were related to expanded disease activity as measured by the European consensus lupus activity measurement, and CH50 levels [24].

In a survey done by Cherica et al., 20% of the respondents with lupus and RA experienced moderate to severe psychological impact. There was moderate to severe anxiety in 38.7% and moderate to severe depression in 27% [25]. Regarding RA patients in this study, 15.5% of RA who found difficulty in getting HCQ had severe anxiety compared to 4.7% of RA patients that did not face this problem.

Our patients with SLE and RA are vulnerable group of patients due to their immunosuppressed status [26]. One other factor that may have contributed to the patients’ anxieties was the clashing reports on the effects (advantageous to hurtful) of hydroxychloroquine in COVID-19 patients [27]. In this study we evaluated factors associated with anxiety, and we observed that anxiety was significantly associated with HCQ shortage, the duration of shortage, CDAI, and SELENA.

HCQ shortage did not adversely affect our patient’s disease activity but lead to psychological impact on our patients, Knowing the psychological status of our patients and the factors influencing their mental status, thus, has provided insight and better understanding of their needs and how best to assist them during these troublesome time

## Conclusion

COVID-19 pandemic resulted in a lack of hydroxychloroquine supplies to the majority of RA and SLE patients, which did not adversely impact their disease activity due to presence of other drug alternatives. However, HCQ shortage was a risk factor for anxiety in those patients. Health care facilities should add HCQ to be a prescription medication to avoid unreasonable drug shortage.

## Data Availability

The data will be available upon reasonable request.

## Abbreviations

RA: Rheumatoid arthritis
SLE: Systemic lupus erythematosus
HCQ: Hydroxychloroquine
CQ: Chloroquine
MHC: Major histocompatibility
COVID-19: Coronavirus disease-19
ACR: American College of Rheumatology
ESR: Erythrocyte Sedimentation Rate
CDAI: Clinical Disease Activity Index
SLEDAI: Systemic Lupus Disease Activity Index
